# The prevalence of chronic medication therapy problems and pharmacists’ interventions among hospitalized perioperative patients: a retrospective observational study

**DOI:** 10.1186/s12913-022-08897-0

**Published:** 2022-12-06

**Authors:** Hai-Ting Cheng, Ming Zhao, Hong-Tao Liu, Guo-Liang Shen, Ting Zhao, Zhi-En Feng

**Affiliations:** 1 Department of Pharmacy, Beijing Stomatological Hospital, Capital Medical University, Beijing, 100050 China; 2 Department of Pharmacy, Beijing Hospital; National Center of Gerontology; Institute of Geriatric Medicine, Chinese Academy of Medical Sciences; Beijing Key Laboratory of Assessment of Clinical Drugs Risk and Individual Application (Beijing Hospital), Beijing, 100730 China; 3 Department of Oral and Maxillofacial-Head and Neck Oncology, Beijing Stomatological Hospital, Capital Medical University, 100050 Beijing, China

**Keywords:** Pharmacists, Drug-related problems, Chronic disease, PCNE classification, Oral and maxillofacial surgery

## Abstract

**Background:**

Inadequate preoperative management of chronic medications can place perioperative patients at risk and cause unnecessary delays in surgical procedures. This study aims to investigate the prevalence of chronic medication therapy problems (CMTPs) in hospitalized perioperative patients and assess the relevance of pharmacists’ interventions.

**Methods:**

We conducted a retrospective study of pharmacist-led preoperative management of chronic medications in hospitalized adult patients from November 2018 to April 2019. The recorded drug-related problems (DRPs) were retrospectively reviewed and categorized according to the Pharmaceutical Care Network Europe classification V9.1 and were analyzed with a multinomial regression model to identify risk factors.

**Results:**

A total of 254 DRPs were recorded, with an average of 0.52 DRPs per patient. Treatment safety (66.9%) was the most common DRP. The most frequent causes of perioperative DRPs and nonperioperative DRPs were drug selection (72.9%) and patient related (50.8%), respectively. Of the 292 documented interventions, 71.6% were fully accepted by the clinicians and patients. The majority (68.9%) of the recorded problems were completely resolved. The number of comorbidities (OR = 3.815) and the number of chronic medications taken (OR = 1.539) were risk factors for the occurrence of DRPs.

**Conclusion:**

The findings of this study suggest that pharmacist-led chronic medication therapy management in surgical wards may be an effective method to help reduce medication-related surgical risks and optimize the medication therapies used for the long-term treatment of chronic diseases.

**Supplementary Information:**

The online version contains supplementary material available at 10.1186/s12913-022-08897-0.

## Background

Half of the perioperative patients have a history of taking chronic medications [1]. Many studies have shown that inadequate preoperative management of chronic medications not only places patients at risk but also causes unnecessary delays in surgical procedures [1–4]. As a result, the potential risks of chronic medication during the perioperative period is an important patient safety concern. However, if chronic medication therapy problems (CMTPs) occur, physicians are unavailable for consultation in the stomatological specialist hospitals. Based on the published references, pharmacists are ideal members of the medical team to support the multi-disciplinary care team with a focus on the identification and resolution of drug-related problems (DRPs) to improve patient outcomes by performing pharmaceutical care activities, such as conducting medication reconciliation, reviewing medication orders, and providing patient education and counseling [5–7]. Therefore, as members of the preoperative team, clinical pharmacists may play an important role in chronic medication therapy management.

Many studies have documented the positive effect of pharmacy services on patients with chronic diseases in different settings, and some of these findings have supported the active involvement of pharmacists to optimize the medication management of perioperative patients [6–9]. Previous studies have focused on medication reconciliation and the DRPs of the medication orders in surgical wards [6, 7, 10, 11]. Very few studies have been conducted on the characteristics of CMTPs in surgical wards and the outcomes of the pharmacists’ interventions.

A DRP is defined as “an event or circumstance involving drug therapy that actually or potentially interferes with desired health outcomes” [12]. The Pharmaceutical Care Network Europe (PCNE) classification for DRPs V9.1 is an established system that has been revised several times in order to be a validated tool to categorize DRPs in multiple settings [5, 13, 14]. It differs from other systems because it separates the problems from the causes.

In this study, we aimed to evaluate the significance of pharmacist-led chronic medication therapy management for hospitalized perioperative patients as well as to assess risk factors for the occurrence of DRPs.

## Methods

### Study design and setting

This retrospective observational study was conducted in a tertiary teaching stomatological hospital in China. The electronic medical records and clinical pharmacist’s ward round records were evaluated. The inclusion criteria were all adult patients admitted to the oral and maxillofacial surgical wards of Beijing Stomatological Hospital, Capital Medical University from November 2018 to April 2019. Patients who were admitted multiple times during the study period were considered another patient at each admission since different DRPs may be experienced during each hospitalization. Patients with incomplete records were excluded for the following reasons: (1) patients whose medication history within 48 h of admission was unknown due to communication barriers or the pharmacist being unable to reach them within 48 h for various reasons, which caused missing information and a failure to intervene in a timely manner, or (2) patients who were discharged within 72 h before the pharmacists provided the intervention.

### Description of the recommendation process used by the clinical pharmacists

The inpatients received care from multidisciplinary teams that included clinical pharmacists who provided pharmaceutical care on weekdays. A clinical pharmacist used the Patient Health Information Form (see the Supplementary Material 1) to collect and document the patients’ information through face-to-face interviews. The form was designed for the patients treated in the Department of Oral and Maxillofacial Surgery [15]. The clinical pharmacists reviewed chronic medication therapy and provided suitable interventions. Patient follow-up visits were conducted during hospitalization. Potential or manifested CMTPs that may have influenced the surgical outcomes or the treatment of chronic diseases were identified. Potential problems were those that were likely to occur, and manifested problems were those that had already occurred. The CMTPs and their possible causes were recorded with reference to the current guidelines [16–20], established literature [21, 22], and standardized databases such as MCDEX^®^, Lexicomp^®^, and UpToDate^®^. Only the drugs classified in the 2019 updated Beers criteria [21] as “medications that are potentially inappropriate in most older adults” were recorded as DRPs in patients aged 65 years and older. The DRPs that were identified, the interventions that were proposed, the acceptance of the interventions and the outcomes were recorded in the clinical pharmacist’s ward round records during the observation period. In this study, the final decisions were made by two clinical pharmacists through discussion. One pharmacist actively worked on the ward and another pharmacist provided consultations. Both of the clinical pharmacists had obtained clinical pharmacy training certificates from the China National Health Commission and had hospital pharmacy experience for longer than six years.

### Data collection and extraction

We collected data from electronic medical records and the clinical pharmacist’s ward round records. This study was carried out in accordance with the Declaration of Helsinki. Patient names were removed during data collection to protect patient privacy. The collected and extracted demographic and health-related data included: sex, age, diagnosis at admission, personal history, laboratory data, medication history, recorded DRPs, interventions, acceptance, and outcomes. The combinations of drugs were determined according to the number of active ingredients; however, this did not apply to herbal products and dietary supplements. The diagnoses of the patients were classified by the International Classification of Diseases 11th Revision (ICD-11) [23].

### Assessment and classification of DRPs

We retrospectively reviewed and categorized the recorded DRPs based on the PCNE classification V9.1. Regarding the cause category, we deleted “dose selection C3”, “treatment duration C4”, “dispensing C5”, “drug use process C6”, and “patient transfer related C8”, because no problems were recorded for those classes. The DRPs that only occurred in perioperative period (defined as the period from admission to discharge for patients who were hospitalized for surgery in this study) and may have affected the surgical outcomes were coded as “perioperative problems”, while the other DRPs that existed in the process of chronic disease treatment and may have affected the long-term treatment of chronic diseases were coded as “nonperioperative problems”.

### Data analysis

Continuous variables are expressed as the mean ± standard deviation; nominal data are expressed as n (%). Data were analyzed with SPSS (Statistical Package for Social Sciences) version 25.0. Logistic regression was used to determine the association between the presence of DRPs and sex, age, number of comorbidities, and number of chronic medications. An unconditional logistic regression analysis was applied, and variables with *P* < 0.2 were included in the multivariate logistic regression analysis, where the variables were selected by a stepwise procedure. The results of the univariate and multivariate analyses were reported as odds ratios (ORs) with 95% confidence intervals (95% CIs). A value of *P* < 0.05 was considered statistically significant.

## Results

### Patients’ characteristics

A total of 573 adult patients (admissions) were admitted from November 2018 to April 2019; 488 (85.2%) patients were included, 23 of whom were admitted more than once during the study period. Eighty-five patients were excluded from the study due to an indeterminant medication history within 48 h of admission (9.1%) and a hospitalization time of less than 72 h (5.8%). The mean age of the patients was 52.0 years ± 15.0 (range 18–86 years). One hundred ninety-five (40%) of the included patients had been taking chronic medications at the time of admission, 11 of whom had no chronic diseases. The most common chronic medications that the patients were taking on admission included nifedipine (43/195, 22.1%), amlodipine (35/195, 17.9%) and metformin (34/195, 17.4%). Comorbidities (which were defined as chronic illnesses or diseases requiring long-term treatment) were present in 192 (39.3%) patients, 8 of whom took no chronic medications at admission. The most common comorbid diseases were hypertension (140/192, 72.9%), diabetes (64/192, 33.3%) and dyslipidemia (47/192, 24.5%). Details regarding the patient demographics and other clinical characteristics are shown in Table 1.

**Table 1:** The demographic and clinical characteristics of the 488 patients

Characteristics	Frequency (n, %)^a^
Sex	
Male	274 (56.1)
Female	214 (43.9)
Age (y), mean ± SD	52.0 ± 15.0
18–64	371 (76.0)
≥ 65	117 (24.0)
Diagnosis at admission^b^	
Malignant neoplasms of lip, oral cavity or pharynx (2B60-2B68, 2B6A)	163 (33.4)
Diseases or disorders of orofacial complex (DA01, DA04-DA07)	144 (29.5)
Benign non-mesenchymal neoplasms (2E90-2E91)	117 (24.0)
Benign mesenchymal neoplasms (2E80-2E83)	35 (7.2)
In situ neoplasms, except of lymphoid, hematopoietic, central nervous system or related tissues (2E60)	17 (3.5)
Others	12 (2.5)
Number of patients taking chronic medications	195 (40.0)
Number of chronic medication categories	
1	54 (11.1)
2	40 (8.2)
3	33 (6.8)
4	22 (4.5)
≥ 5	46 (9.4)
Most common chronic medications	
Nifedipine	43 (8.8)
Amlodipine	35 (7.2)
Metformin	34 (7.0)
Number of patients having comorbidities	192 (39.3)
Number of comorbidity categories	
1	84 (17.2)
2	59 (12.1)
≥3	49 (10.0)
Most common comorbidities	
Hypertension	140 (28.7)
Diabetes	64 (13.1)
Dyslipidemia	47 (9.6)
Allergies	
No known drug allergy	409 (83.8)
Yes (to one or more medications)	79 (16.2)

*SD* standard deviation

^a^Unless indicated otherwise, all results are presented as frequencies (n, %)

^b^The disease types were categorized according to the WHO-ICD 11

### Details on the DRPs

A total of 254 DRPs were recorded, and an average rate of 0.52 DRPs per patient was calculated. Among the 488 included patients, 147 (30.1%) experienced at least one DRP. In addition, 72.4% of the patients undergoing chronic diseases/treatment (*n* = 203) experienced at least one DRP. As shown in Table 2, the major problems included “treatment safety” (66.9%), followed by “treatment effectiveness” (30.7%) and “other” (2.4%). All of the recorded DRPs were further classified as perioperative (144/254, 56.7%) and nonperioperative (110/254, 43.3%), as well as potential (179/254, 70.5%) and manifested (75/254, 29.5%). Based on the 2019 updated Beers criteria, 7 treatment safety problems were associated with potentially inappropriate medication use. The most commonly inappropriately used medication in older adults (≥ 65 years) was eszopiclone (*n* = 3), followed by reserpine (> 0.1 mg/day) (*n* = 2), nifedipine (immediate release) (*n* = 1), and estazolam (*n* = 1).

**Table 2:** The types of problems, interventions and acceptance of DRPs according to the PCNE classification V9.1

**Code**	**Detailed classification**^**a**^	**Total**	**Perioperative DRPs**	**Nonperioperative DRPs**
		**n (%)**	**n (%)**	**n (%)**
**Problems**		**254 (100)**	**144 (100)**	**110 (100)**
P1	Treatment effectiveness	78 (30.7)	20 (13.9)	58 (52.7)
P1.1	No effect of drug treatment despite correct use	1 (0.4)	-	1 (0.9)
P1.2	Effect of drug treatment not optimal	64 (25.2)	20 (13.9)	44 (40.0)
P1.3	Untreated symptoms or indication	13 (5.1)	-	13 (11.8)
P2	Treatment safety	170 (66.9)	124 (86.1)	46 (41.8)
P2.1	Adverse drug event (possibly) occurring	170 (66.9)	124 (86.1)	46 (41.8)
P3	Other	6 (2.4)	-	6 (5.5)
P3.1	Unnecessary drug-treatment	6 (2.4)	-	6 (5.5)
**Interventions**		**292 (100)**	**154 (100)**	**138 (100)**
I1	At prescriber level	165 (56.5)	143 (92.9)	22 (15.9)
I1.3	Intervention proposed to prescriber	144 (49.3)	132 (85.7)	12 (8.7)
I1.4	Intervention discussed with prescriber	21 (7.2)	11 (7.1)	10 (7.2)
I2	At patient level	110 (37.7)	11 (7.1)	99 (71.7)
I2.1	Patient (drug) counselling	43 (14.7)	11 (7.1)	32 (23.2)
I2.2	Written information provided (only)	24 (8.2)	-	24 (17.4)
I2.3	Patient referred to prescriber	43 (14.7)	-	43 (31.2)
I3	At drug level	16 (5.5)	-	16 (11.6)
I3.4	Instructions for use changed to …	9 (3.1)	-	9 (6.5)
I3.5	Drug paused or stopped	7 (2.4)	-	7 (5.1)
I4	Other intervention or activity	1 (0.3)	-	1 (0.7)
I4.2	Side effect reported to authorities	1 (0.3)	-	1 (0.7)
**Acceptance**		**292 (100)**	**154 (100)**	**138 (100)**
A1	Intervention accepted	285 (97.6)	154 (100)	131 (94.9)
A1.1	Intervention accepted and fully implemented	209 (71.6)	143 (92.9)	66 (47.8)
A1.2	Intervention accepted, partially implemented	1 (0.3)	-	1 (0.7)
A1.3	Intervention accepted but not implemented	10 (3.4)	-	10 (7.2)
A1.4	Intervention accepted, implementation unknown	65 (22.2)	11 (7.1)	54 (39.1)
A2	Intervention not accepted	7 (2.4)	-	7 (5.1)
A2.2	Intervention not accepted: no agreement	7 (2.4)	-	7 (5.1)

*DRPs* drug-related problems, *PCNE* Pharmaceutical Care Network Europe

^a^Only items that have a frequency of more than one were included

The perioperative DRPs were most commonly associated with angiotensin-converting enzyme inhibitors (ACEIs) and angiotensin II receptor blockers (ARBs) (75/144, 52.1%), antiplatelet agents (20/144, 13.9%), and calcium channel blockers (19/144, 13.2%). The nonperioperative DRPs were most commonly associated with insulins (13/110, 11.8%), sulfonylureas (12/110, 10.9%), and herbal products (12/110, 10.9%).

### Causes of DRPs

Each DRP could have more than one cause. A total of 268 causes were recorded; 53.7% were the causes of perioperative DRPs; and 46.3% were the ones of nonperioperative DRPs. The primary cause of perioperative DRPs was “drug selection C1” (72.9%), followed by “drug form C2” (19.4%) and “patient related C7” (7.6%). Regarding the nonperioperative DRPs, the primary cause was “patient related C7” (50.8%), followed by “drug selection C1” (46.0%) and “other C9” (3.2%). Table 3 shows the details and examples of the causes of the perioperative DRPs and nonperioperative DRPs.

**Table 3:** Causes of the DRPs according to the PCNE classification V9.1

**Code**	**Detailed classification**^**a**^	**Perioperative DRPs**		**Nonperioperative DRPs**	
		***n* = 144**		***n* = 124**	
		**n (%)**	**Example (n)**	**n (%)**	**Example (n)**
C1	Drug selection	105 (72.9)		57 (46.0)	
C1.1	Inappropriate drug according to guidelines/formulary	105 (72.9)	Taking ACEI/ARB increased the hypotension risk during the perioperative period (75)	21 (16.9)	Irrational self-medication with herbal products (7)
C1.2	No indication for drug	-	-	6 (4.8)	Taking aspirin as a supplement for health protection (4)
C1.3	Inappropriate combination of drugs, or drugs and herbal medications, or drugs and dietary supplements	-	-	1 (0.8)	The combination of zopiclone and estazolam for insomnia in a 63-year-old patient (1)
C1.5	No or incomplete drug treatment in spite of existing indication	-	-	29 (23.4)	The unknown presence of or incompletely treated diabetes before admission (14)
C2	Drug form	28 (19.4)		-	
C2.1	Inappropriate drug form/formulation (for this patient)	28 (19.4)	Taking nifedipine controlled-release tablets that are unsuitable for nasogastric tube administration (18)	-	-
C7	Patient related	11 (7.6)		63 (50.8)	
C7.1	Patient intentionally uses/takes less drug than prescribed or does not take the drug at all for whatever reason	11 (7.6)	Self-discontinuation of chronic medications before admission for fear of affecting the surgery and preoperative examinations (11)	25 (20.2)	Poor adherence due to forgetfulness or fear of adverse drug reactions (23)
C7.5	Patient takes food that interacts	-	-	1 (0.8)	Drinking a lot of porridge every day which leads to poor glucose control (1)
C7.6	Patient stores drug inappropriately	-	-	8 (6.5)	Keeping the in-use insulin pen along with the needle refrigerated (2 to 8 °C) (8)
C7.7	Inappropriate timing or dosing intervals	-	-	18 (14.5)	Inappropriate time of taking diabetes medications (9)
C7.8	Patient unintentionally administers/uses the drug in a wrong way	-	-	10 (8.1)	No resuspending before using the premixed insulin pen (1).
C7.9	Patient physically unable to use drug/form as directed	-	-	1 (0.8)	Cannot split the small tablets of medications in half (1).
C9	Other	-		4 (3.2)	
C9.1	No or inappropriate outcome monitoring (incl. TDM)	-	-	4 (3.2)	The self-monitoring of blood glucose not including a postprandial 2 h glucose (1)

*DRPs* drug-related problems, *PCNE* Pharmaceutical Care Network Europe, *ACEI* angiotensin-converting enzyme inhibitor, *ARB* angiotensin II receptor blocker, *TDM* therapeutic drug monitoring

^a^Only items that had a frequency of more than one were included

### Interventions and acceptance

Each DRP could have more than one intervention. In total, 292 documented interventions were proposed by the clinical pharmacists with the aim of resolving the recorded DRPs. The most common interventions that were proposed to resolve the perioperative DRPs were made at the prescriber level (92.9%), while the interventions that were proposed to resolve the nonperioperative DRPs were mainly made at the patient level (71.7%). The majority (71.6%) of the interventions were accepted and fully implemented by the clinicians or patients, followed by interventions that were accepted with an unknown implementation status (22.2%). Table 2 shows an explanation of interventions and acceptance.

### Outcomes of the interventions

Among the 254 recorded DRPs, the main outcomes of interventions were “solved O1” (68.9%), followed by “not known O0” (14.2%), “partially solved O2” (13.0%) and “not solved O3” (3.9%). Regarding the perioperative DRPs, 94 potential DRPs, which might have led to a risk of either perioperative hypertension or hypotension, were prevented in patients with hypertension; 23 potential DRPs, which might have caused a risk of perioperative hyperglycemia or hypoglycemia, were successfully prevented in patients with diabetes; and 14 potential DRPs, which might have led to excessive bleeding or thromboembolic complications, were prevented in patients who were taking anticoagulants, antiplatelet drugs or herbal products, such as ginkgo and ginseng, on admission. Regarding the nonperioperative DRPs, the totally solved DRPs were mostly “adverse drug event possibly occurring” (21/42, 50.0%). The majority of the partially solved DRPs and the DRPs with an unknown status were the “effect of drug treatment not optimal” (15/22, 68.2%; 15/36, 41.7%, respectively), and the unsolved DRPs were mostly “adverse drug event possibly occurring” (6/10, 60.0%), due to a lack of patient cooperation. Detailed descriptions of the outcomes of the documented interventions for the perioperative DRPs and the nonperioperative DRPs are provided in Tables 4 and 5, respectively.

**Table 4:** Outcomes of the pharmacists’ interventions on the perioperative DRPs according to the PCNE classification V9.1

Perioperative risks	Total DRPs	Totally solved	Partially solved
	**n**	**n (%)**	**n (%)**
Hypertension/hypotension	96	94 (97.9)^a^	2 (2.1)
Hyperglycemia/hypoglycemia	23	23 (100)^a^	-
Hemorrhagic/thrombotic	21	14 (66.7)^a^	7 (33.3)
Gastrointestinal discomfort	2	2 (100)^a^	-
Dyslipidemia	2	-	2 (100)
**Total**	**144**	**133 (92.4)** ^**a**^	**11 (7.6)**

*DRPs* drug-related problems

^a^Potential problem

**Table 5:** Outcomes of the pharmacists’ interventions for the nonoperative DRPs according to the PCNE classification V9.1

**DRPs classification**	**Total**	**Totally resolved**	**Partially resolved**	**Not solved, lack of cooperation of the patient**	**Status unknown**
	**n**	**n (%)**	**n (%)**	**n (%)**	**n (%)**
No effect of drug treatment	1	1 (100)	-	-	-
Effect of drug treatment not optimal	44	12 (27.3)	15 (34.1)	2 (4.5)	15 (34.1)
Untreated symptoms or indication	13	4 (30.8)	-	-	9 (69.2)
Adverse drug event possibly occurring^a^	44	21 (47.7)	6 (13.6)	6 (13.6)	11 (25.0)
Adverse drug event occurring	2	1 (50.0)	-	-	1 (50.0)
Unnecessary drug-treatment	6	3 (50.0)	1 (16.7)	2 (33.3)	-
**Total**	**110**	**42 (38.2)**	**22 (20.0)**	**10 (9.1)**	**36 (32.7)**

*DRPs* drug-related problems

^a^Potential problem

### Potential risk factors for the occurrence of DRPs

The results of the logistic regression analysis are shown in Table 6. The univariate analysis showed that sex, age, the number of comorbidities and the number of chronic medications all had *P* values of < 0.2. These factors were analyzed in the full model. The results indicated that the number of comorbidities and number of chronic medications were statistically significant (*P* < 0.05), the ORs were 3.815 and 1.539, and the corresponding 95% CIs were 2.297, 6.337 and 1.153, 2.055, respectively.

**Table 6:** Factors associated with the occurrence of DRPs

**Factors**	**Univariate**		**Multivariate**	
	**OR (95% CI)**	***P*** **value**	**OR (95% CI)**	***P*** **value**
Sex	1.294 (0.873–1.918)	0.199	-	-
Age	1.060 (1.044–1.077)	< 0.001	-	-
Number of comorbidities	6.998 (4.965–9.865)	< 0.001	3.815 (2.297–6.337)	< 0.001
Number of chronic medications (per active increase)	3.035 (2.471–3.729)	< 0.001	1.539 (1.153–2.055)	0.003

*DRPs* drug-related problems, *OR* odds ratio, *CI* confidence interval

## Discussion

To the best of the authors’ knowledge, this was the first retrospective study that has been conducted to categorically evaluate CMTPs among perioperative patients at a Chinese stomatological hospital and to analyze the utility of pharmacist interventions. In our hospital, the inpatients were required to bring their own chronic medications at admission, and, the preanesthetic evaluation generally took place before the day of surgery, which raises the importance of using the knowledge of pharmacists for chronic medication therapy management. This study showed that pharmacist-led chronic medication therapy management significantly prevented the occurrence of potential DRPs and solved the DRPs that developed.

In our study, 30.1% of patients experienced at least one DRP, and the average rate of DRPs per patient was 0.52, which differed from the results of previous studies using the PCNE classification to evaluate DRPs [24, 25]. An analysis of hospitalized surgical patients reported that 19.6% of the patients included in that study had DRPs and that there was an average of 0.3 DRPs per patient [24]. Another study found that, in the oncology ward, 83.2% of the patients had DRPs and that there was an average occurrence of 3.5 DRPs per patient [25]. There might be several explanations for the difference of the number of DRPs that were observed in this study compared to the results of other studies. First, our study was conducted in a stomatological hospital rather than in a general hospital. In China, because physicians are unavailable for consultation in the stomatological hospitals, generally, only patients in good physical condition are hospitalized for oral or maxillofacial surgery. Second, our study focused on CMTPs without analyzing the medication orders related to surgery. In addition, our study reported that at least one DRP was detected in 72.4% of the patients with chronic diseases/treatment, highlighting the need for clinical pharmacists to help with chronic medication management in the perioperative period.

The possible occurrence of adverse drug events were the most common preoperative DRPs in this study, and this was consistent with the results of a previous study [11]. It is well known that perioperative patients who take chronic medications are at high risk of complications. For example, in this study, the patients who underwent free-flap reconstruction surgery required nasal feeding for several days after surgery. However, nifedipine, the most common chronic medication used in this study, is often available in controlled-release formulations, and it may lead to hypotension if it is crushed before administration. Moreover, ACEIs and ARBs should be withheld on the morning of surgery based on concerns about possible hypotension. The clinical pharmacists should consult with the surgeons to determine which chronic medications should be stopped or replaced, and alternative drugs should be recommended to reduce the occurrence of preoperative risks associated with CMTPs.

In this study, the clinical pharmacists not only focused on the preoperative DRPs but also actively intervened in the nonpreoperative DRPs. The “effect of drug treatment not optimal” and “adverse drug event possibly occurring” were the two most frequent problem subtypes of nonpreoperative DRPs. In this study, it was found that many patients with poor glycemic control lacked awareness of the importance of proper nutrition and exercise, and used or stored insulin pens incorrectly. The high incidence of lack of effectiveness and the safety problems in this study highlighted the need for clinical pharmacists to support hospitalized patients in the self-management of chronic diseases.

For perioperative DRPs, the high acceptance rate (92.9%) of the interventions and the improved outcomes could be explained by the following facts. First, the clinical pharmacists who were actively working on the ward received a large amount of professional clinical pharmacy training (including a 3-year general pharmacy practice residency, a 2-year specialized residency and a 1-year clinical pharmacist training). Second, after more than three years of cooperation, the surgeons were very willing to let the clinical pharmacist join the treatment team. Third, the solutions to most perioperative DRPs are generally clear. In addition, the acceptance rate of the interventions for nonperioperative DRPs was 47.8%, which was lower than that in other studies [8, 25]. This was mainly caused by the fact that despite 39.1% of the interventions being accepted, the implementation status was unknown, which mainly had to do with whether the patients visited doctors or whether the patient adherence was improved after discharge.

The multivariate analysis showed that the number of comorbidities and the number of chronic medications taken were risk factors for the occurrence of DRPs, and this was consistent with the results from other studies [26, 27]. Therefore, clinical pharmacists should closely monitor the patients with multiple comorbidities or polypharmacy. In our study, age and sex were not risk factors associated with the development of DRPs, however, previous findings were inconsistent. A study on patients with cardiovascular diseases found that age and sex were not risk factors for DRPs [28]. However, Mateti et al. [29] revealed that age and female sex were risk factors for developing DRPs.

This study has some limitations. First, this research did not involve the process of prescribing and dispensing of the chronic medications. Thus, some DRPs may have been missed due to a lack of necessary information. Second, as this study was conducted in a stomatological specialist hospital, the results may not be generalizable to other hospitals. Finally, a major limitation is our inability to determine the effects of the interventions on health outcomes due to the retrospective observational nature of our study, lack of a control group and the lack of patient information after discharge.

The findings of this study promoted involvement of clinical pharmacists into the multidisciplinary team to improve chronic medication safety during the perioperative period, which are essential for the development and implementation of clinical pharmacist-led chronic medication therapy management in surgical wards in China. The effect of a clinical pharmacist-led chronic medication therapy management on the health outcomes of hospitalized perioperative patients could be evaluated in future randomized controlled trials with long-term follow-up.

## Conclusion

In this study, CMTPs were commonly observed among perioperative patients with chronic diseases/treatment. Based on our findings, pharmacist-led chronic medication therapy management significantly prevented the occurrence of potential DRPs that may have affected the surgical outcomes and solved some of the DRPs that may have affected the long-term treatment of chronic diseases among preoperative patients. These findings suggest that pharmacist-led chronic medication therapy management in the surgical ward may be an effective method to help reduce medication-related surgical risks and optimize the medication therapies used for the long-term treatment of chronic diseases among preoperative patients.

## Supplementary Information

**Additional file 1.**

## Data Availability

The datasets generated during the study and/or analyzed during the current study are available from the corresponding author upon reasonable request.

## Abbreviations

CMTP: Chronic medication therapy problem
DRP: Drug-related problem
PCNE: Pharmaceutical Care Network Europe
ICD-11: International Classification of Diseases 11th Revision
SPSS: Statistical Package for Social Sciences
OR: Odds ratio
CI: Confidence interval
WHO: World Health Organization
ACEI: Angiotensin-converting enzyme inhibitor
ARB: Angiotensin II receptor blocker
TDM: Therapeutic drug monitoring
