# Supplementary material for: The prevalence of chronic medication therapy problems and pharmacists’ interventions among hospitalized perioperative patients: a retrospective observational study
Source: BMC Health Serv Res. 2022 Dec 6;22:1483. doi: 10.1186/s12913-022-08897-0 (PMC9724277; doi:10.1186/s12913-022-08897-0)
Supplement: Supplementary file 1 — Additional file 1. [file 12913_2022_8897_MOESM1_ESM.docx]

**Supplementary Material 1**

| **Patient Health Information Form** | | | | | | | | | | | | | | | | | | | | | |
| --- | --- | --- | --- | --- | --- | --- | --- | --- | --- | --- | --- | --- | --- | --- | --- | --- | --- | --- | --- | --- | --- |
| Name: | | | | Medical Record Number: | | | | | Bed Number: | | | | | | Sex: | | | Age: | | Race: | |
| Date of Admission: | | | | | | | | | | | | Date of Discharge: | | | | | | | | | |
| Admission Diagnosis: 1. , 2. , 3. , … | | | | | | | | | | | | | | | | | | | | | |
| Planned Operation: | | | | | | | | | | | | | | | | | | | | | |
| **Family Health History** | | | | | | | | | | | | | | | | | | | | | |
| Hypertension | Diabetes | | | Dyslipidemia | | Asthma | | Stroke | | | Depression | | | | | Other: | | | | | |
| **Medical History** | | | | | | | | | | | | | | | | | | | | | |
| Hypertension  Depression | Diabetes  Gout | | | Dyslipidemia  Stroke | | Asthma  Insomnia | | Coronary Heart Disease  Gastroesophageal Reflux Disease | | | | | | | | Chronic Obstructive Pulmonary Disease  Other: | | | | | |
| **Surgery History** | | | | | | | | | | | | | | | | | | | | | |
| Oral and Maxillofacial Surgery | | | Coronary Artery Bypass Grafting | | | | | Percutaneous Coronary Intervention | | | | | | | | Other: | | | | | |
| **Current Medications (prescription and nonprescription** **medications, herbal products, dietary supplements)** | | | | | | | | | | | | | | | | | | | | | |
| Name | | Dose | | Take for… | | | When do you take the medications? | | | | | | | Start Date | | | Stop Date | | How did you get the medications? | | |
|  |  |  |  |  |  |  | Morning | Noon | | Night | | | Bedtime |  |  |  |  |  | Physician prescribed | | Self-medication |
|  | |  | |  | | |  |  | |  | | |  |  | | |  | | **** | |  |
|  | |  | |  | | |  |  | |  | | |  |  | | |  | | **** | |  |
|  | |  | |  | | |  |  | |  | | |  |  | | |  | | **** | |  |
|  | |  | |  | | |  |  | |  | | |  |  | | |  | | **** | | **** |
|  | |  | |  | | |  |  | |  | | |  |  | | |  | | **** | |  |
| Have you ever omitted drugs?  Yes No; How often? ; Why? . | | | | | | | | | | | | | | | | | | | | | |
| Have you ever borrowed drugs from others?  Yes No; How often? ; Why? . | | | | | | | | | | | | | | | | | | | | | |
| **Adverse Reactions Caused by** **Drugs or Food** | | | | | **Event Description** | | | | | | | | | | | | | | | | |
|  | | | | |  | | | | | | | | | | | | | | | | |
